# 12-Benzoyl-2-methyl­naphtho­[2,3-*b*]indolizine-6,11-dione

**DOI:** 10.1107/S1600536811019623

**Published:** 2011-05-28

**Authors:** Yun Liu, Su-Hui Wang, Shu-Ren Shen, Zong-Hui Yang

**Affiliations:** a School of Chemistry and Chemical Engineering, Xuzhou Normal University, Xuzhou, Jiangsu 221116, People’s Republic of China

## Abstract

In the title compound, C_24_H_15_NO_3_, the fused naphthaquin­one–pyrrole unit is approximately planar, the naphthaquinone ring system making a dihedral angle of 2.91 (10)° with the pyrrole ring. The plane of the pyrrole ring makes a dihedral angle 61.64 (14)° with that of the benzene ring of the benzoyl­methyl­ene group. The crystal structure is stablized by intra­molecular C—H⋯O inter­actions.

## Related literature

For the properties of indolizine, see Olden *et al.* (1991 ▶); Jaffrezou *et al.* (1992 ▶). For the preparation of benzo[*f*]pyrido[1,2-*a*]indole-6,11-dione, see Pratt *et al.* (1957 ▶). For bond-length data, see: Allen *et al.* (1987 ▶).
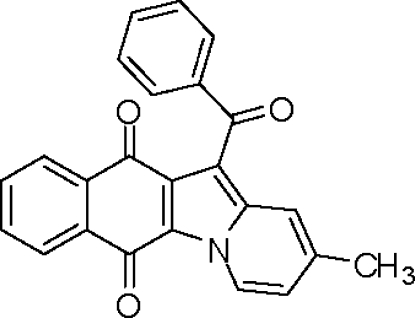


## Experimental

### 

#### Crystal data

C_24_H_15_NO_3_
*M* *_r_* = 365.37
Monoclinic, 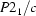

*a* = 7.1260 (14) Å
*b* = 10.125 (2) Å
*c* = 24.352 (5) Å
β = 90.22 (3)°
*V* = 1757.0 (6) Å^3^
*Z* = 4
Mo *K*α radiation
μ = 0.09 mm^−1^
*T* = 295 K
0.30 × 0.20 × 0.10 mm

#### Data collection

Enraf–Nonius CAD-4 diffractometer
Absorption correction: ψ scan (*XCAD4*; Harms & Wocadlo, 1995 ▶) *T*_min_ = 0.973, *T*_max_ = 0.991
3371 measured reflections
3103 independent reflections
1555 reflections with *I* > 2σ(*I*)
*R*_int_ = 0.062
3 standard reflections every 200 reflections  intensity decay: none

#### Refinement

*R*[*F* ^2^ > 2σ(*F* ^2^)] = 0.064
*wR*(*F* ^2^) = 0.169
*S* = 1.02
3103 reflections
254 parameters
H-atom parameters constrained
Δρ_max_ = 0.21 e Å^−3^
Δρ_min_ = −0.22 e Å^−3^

### 

Data collection: *CAD-4 Software* (Enraf–Nonius, 1989 ▶); cell refinement: *CAD-4 Software*; data reduction: *XCAD4* (Harms & Wocadlo, 1995 ▶); program(s) used to solve structure: *SHELXS97* (Sheldrick, 2008 ▶); program(s) used to refine structure: *SHELXL97* (Sheldrick, 2008 ▶); molecular graphics: *SHELXTL* (Sheldrick, 2008 ▶); software used to prepare material for publication: *SHELXTL* and *PLATON* (Spek, 2009 ▶).

## Supplementary Material

Crystal structure: contains datablocks I, global. DOI: 10.1107/S1600536811019623/ds2112sup1.cif

Structure factors: contains datablocks I. DOI: 10.1107/S1600536811019623/ds2112Isup2.hkl

Supplementary material file. DOI: 10.1107/S1600536811019623/ds2112Isup3.cml

Additional supplementary materials:  crystallographic information; 3D view; checkCIF report

## Figures and Tables

**Table 1:** Hydrogen-bond geometry (Å, °)

*D*—H⋯*A*	*D*—H	H⋯*A*	*D*⋯*A*	*D*—H⋯*A*
C10—H10⋯O3^i^	0.93	2.45	3.305 (5)	152

Symmetry code: (i) 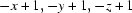.

**Fig. 1.**
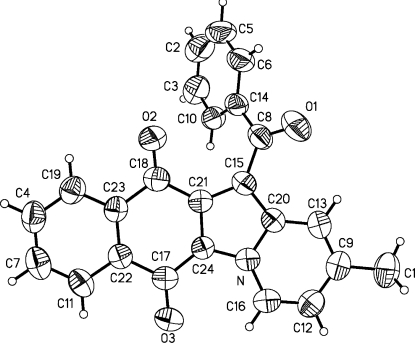
The molecular structure of (I), with atom labels and 50% probability displacement ellipsoids for non-H atoms.

**Fig. 2.**
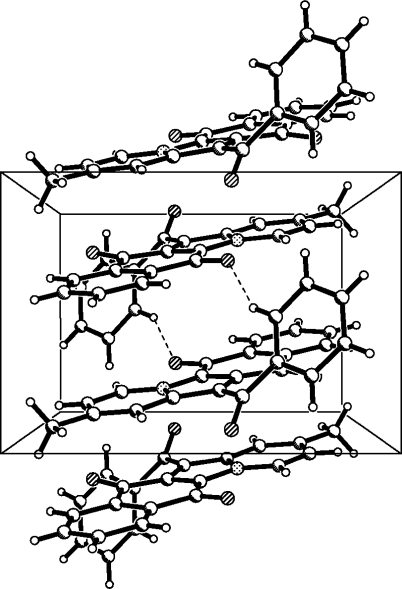
The crystal packing of the title compound, viewed along the c axis.

## supplementary crystallographic information

### Comment

The natural and many synthetic indolizines have a diversity of biological activity and are playing an increasingly important role in developing new pharmaceuticals [Olden *et al.*, 1991; Jaffrezou *et al.*, 1992]. Benzo[*f*]pyrido[1,2-*a*]indole-6,11-diones are benzo-fused indolizines and occur in several marine alkaloids. The synthesis of these compounds has drawn much research interest [Pratt *et al.*, 1957]. In our ongoing research work on the direct one pot syntheses of benzo[*f*]pyrido[1,2-*a*]indole-6,11-diones, we have prepared the title compound (I). As part of this study, we have undertaken an X-ray crystallographic analysis of (I) in order to confirm its structure.

The bond lengths and angles of the title molecule (Fig. 1) are within normal ranges (Allen *et al.*, 1987). The naphthaquinone ring is essentially planar to the pyrrole ring with the dihedral angel being 2.91 (10)°. The pyrrole ring makes the dihedral angle 61.64 (14)° with the benzene ring of the benzoylmethylene group. Although atoms C16, C20 and C24 attached to atom N are all of *sp*^2^ hybridization, their different environments cause slight differences in the N—C16, N—C20 and N—C24 bond lengths, and in the C16—N—C20, C16— N—C24, and C20—N—C24 angles (Table 1). The molecular packing is stabilized by weak intermolecular C—H···O hydrogen bonds.

### Experimental

The compound (I) was prepared by the reaction of 4-methyl pyridine (1.0 mmol), benzoylacetone (1.0 mmol), and 2,3-dichloro-1,4-naphthaquionone (1.0 mmol) mixed in 10 mL CH3CN. The reaction mixture were heated to reflux for 24 h and was isolated by column chromatography after evaporation of the solvent. Single crystals of (I) were obtained by slow evaporation from an petroleum ether-ethyl acetate(3:1) solvent system (yield 62%).

### Refinement

The H atoms were geometrically placed and were treated as riding, with C—H = 0.93 Å

### Crystal data

**Table:**

C_24_H_15_NO_3_	*F*(000) = 760
*M**_r_* = 365.37	*D*_x_ = 1.381 Mg m^−^^3^
Monoclinic, *P*2_1_/*c*	Melting point: 538 K
Hall symbol: -P 2ybc	Mo *K*α radiation, λ = 0.71073 Å
*a* = 7.1260 (14) Å	Cell parameters from 25 reflections
*b* = 10.125 (2) Å	θ = 9–12°
*c* = 24.352 (5) Å	µ = 0.09 mm^−^^1^
β = 90.22 (3)°	*T* = 295 K
*V* = 1757.0 (6) Å^3^	Block, red
*Z* = 4	0.30 × 0.20 × 0.10 mm

### Data collection

**Table:**

Enraf–Nonius CAD-4 diffractometer	1555 reflections with *I* > 2σ(*I*)
Radiation source: fine-focus sealed tube	*R*_int_ = 0.062
graphite	θ_max_ = 25.0°, θ_min_ = 1.7°
ω/2θ scans	*h* = −8→0
Absorption correction: ψ scan (*XCAD4*; Harms & Wocadlo, 1995)	*k* = −12→0
*T*_min_ = 0.973, *T*_max_ = 0.991	*l* = −28→28
3371 measured reflections	3 standard reflections every 200 reflections
3103 independent reflections	intensity decay: none

### Refinement

**Table:**

Refinement on *F*^2^	Primary atom site location: structure-invariant direct methods
Least-squares matrix: full	Secondary atom site location: difference Fourier map
*R*[*F*^2^ > 2σ(*F*^2^)] = 0.064	Hydrogen site location: inferred from neighbouring sites
*wR*(*F*^2^) = 0.169	H-atom parameters constrained
*S* = 1.02	*w* = 1/[σ^2^(*F*_o_^2^) + (0.0675*P*)^2^ + 0.0651*P*] where *P* = (*F*_o_^2^ + 2*F*_c_^2^)/3
3103 reflections	(Δ/σ)_max_ = 0.006
254 parameters	Δρ_max_ = 0.21 e Å^−^^3^
0 restraints	Δρ_min_ = −0.22 e Å^−^^3^

### Special details

**Table:**

Geometry. All e.s.d.'s (except the e.s.d. in the dihedral angle between two l.s. planes) are estimated using the full covariance matrix. The cell e.s.d.'s are taken into account individually in the estimation of e.s.d.'s in distances, angles and torsion angles; correlations between e.s.d.'s in cell parameters are only used when they are defined by crystal symmetry. An approximate (isotropic) treatment of cell e.s.d.'s is used for estimating e.s.d.'s involving l.s. planes.
Refinement. Refinement of *F*^2^ against ALL reflections. The weighted *R*-factor *wR* and goodness of fit *S* are based on *F*^2^, conventional *R*-factors *R* are based on *F*, with *F* set to zero for negative *F*^2^. The threshold expression of *F*^2^ > σ(*F*^2^) is used only for calculating *R*-factors(gt) *etc*. and is not relevant to the choice of reflections for refinement. *R*-factors based on *F*^2^ are statistically about twice as large as those based on *F*, and *R*- factors based on ALL data will be even larger.

### Fractional atomic coordinates and isotropic or equivalent isotropic displacement parameters (Å^2^)

**Table:**

	*x*	*y*	*z*	*U*_iso_*/*U*_eq_	
N	0.1808 (3)	0.6115 (3)	0.44928 (11)	0.0478 (7)	
C24	0.2311 (4)	0.4972 (3)	0.47630 (13)	0.0446 (8)	
O3	0.2792 (4)	0.5789 (3)	0.56513 (10)	0.0699 (8)	
O2	0.2339 (4)	0.1643 (3)	0.42739 (10)	0.0806 (9)	
C23	0.3188 (4)	0.2429 (3)	0.51506 (14)	0.0492 (9)	
C22	0.3265 (4)	0.3501 (3)	0.55128 (14)	0.0494 (9)	
C21	0.2309 (4)	0.3959 (3)	0.43837 (13)	0.0470 (8)	
C20	0.1497 (4)	0.5816 (3)	0.39425 (14)	0.0490 (9)	
C19	0.3601 (5)	0.1177 (4)	0.53431 (16)	0.0630 (10)	
H19	0.3511	0.0459	0.5107	0.076*	
C18	0.2598 (5)	0.2599 (4)	0.45665 (15)	0.0571 (10)	
C17	0.2775 (5)	0.4848 (4)	0.53324 (14)	0.0508 (9)	
C16	0.1643 (5)	0.7379 (4)	0.46870 (15)	0.0553 (9)	
H16	0.1848	0.7557	0.5057	0.066*	
C15	0.1807 (5)	0.4458 (4)	0.38678 (13)	0.0525 (9)	
C14	0.3024 (6)	0.2844 (3)	0.31344 (13)	0.0572 (10)	
O1	0.0328 (4)	0.4141 (3)	0.30306 (11)	0.0967 (10)	
C13	0.1000 (5)	0.6856 (4)	0.35921 (15)	0.0594 (10)	
H13	0.0775	0.6684	0.3223	0.071*	
C12	0.1184 (5)	0.8354 (4)	0.43411 (17)	0.0633 (11)	
H12	0.1092	0.9213	0.4475	0.076*	
C11	0.3777 (5)	0.3285 (4)	0.60562 (14)	0.0621 (10)	
H11	0.3820	0.3988	0.6302	0.075*	
C10	0.4852 (6)	0.2881 (4)	0.33161 (15)	0.0648 (11)	
H10	0.5208	0.3493	0.3582	0.078*	
C9	0.0840 (5)	0.8116 (4)	0.37831 (17)	0.0617 (10)	
C8	0.1615 (5)	0.3819 (4)	0.33307 (15)	0.0611 (10)	
C7	0.4220 (5)	0.2029 (5)	0.62324 (17)	0.0725 (12)	
H7	0.4577	0.1894	0.6596	0.087*	
C6	0.2519 (7)	0.1918 (4)	0.27499 (15)	0.0780 (13)	
H6	0.1291	0.1897	0.2619	0.094*	
C5	0.3799 (9)	0.1024 (5)	0.25553 (18)	0.0969 (17)	
H5	0.3424	0.0377	0.2307	0.116*	
C4	0.4142 (5)	0.0979 (4)	0.58804 (17)	0.0733 (12)	
H4	0.4453	0.0137	0.6003	0.088*	
C3	0.6168 (7)	0.2010 (4)	0.31047 (18)	0.0839 (13)	
H3	0.7413	0.2054	0.3219	0.101*	
C2	0.5615 (9)	0.1081 (5)	0.2725 (2)	0.0987 (17)	
H2	0.6488	0.0491	0.2583	0.118*	
C1	0.0376 (6)	0.9245 (4)	0.34044 (18)	0.0885 (14)	
H1A	0.0216	0.8917	0.3037	0.133*	
H1B	0.1379	0.9877	0.3412	0.133*	
H1C	−0.0765	0.9660	0.3523	0.133*	

### Atomic displacement parameters (Å^2^)

**Table:**

	*U*^11^	*U*^22^	*U*^33^	*U*^12^	*U*^13^	*U*^23^
N	0.0436 (17)	0.0484 (18)	0.0514 (18)	−0.0055 (14)	0.0046 (13)	−0.0026 (15)
C24	0.0354 (18)	0.048 (2)	0.050 (2)	0.0007 (16)	0.0012 (16)	−0.0009 (18)
O3	0.083 (2)	0.0695 (18)	0.0574 (16)	−0.0013 (15)	−0.0041 (13)	−0.0141 (14)
O2	0.123 (3)	0.0532 (17)	0.0652 (17)	−0.0080 (16)	−0.0049 (16)	−0.0040 (14)
C23	0.0345 (19)	0.055 (2)	0.058 (2)	0.0068 (17)	0.0040 (15)	0.0040 (19)
C22	0.037 (2)	0.059 (2)	0.051 (2)	0.0021 (17)	0.0061 (16)	0.0020 (19)
C21	0.040 (2)	0.049 (2)	0.052 (2)	−0.0050 (17)	0.0013 (16)	−0.0032 (18)
C20	0.039 (2)	0.058 (2)	0.050 (2)	−0.0035 (17)	0.0000 (16)	0.0002 (19)
C19	0.056 (2)	0.061 (3)	0.072 (3)	0.010 (2)	0.004 (2)	0.003 (2)
C18	0.058 (2)	0.058 (2)	0.055 (2)	−0.008 (2)	0.0059 (18)	−0.003 (2)
C17	0.041 (2)	0.056 (2)	0.055 (2)	−0.0032 (17)	0.0051 (17)	−0.005 (2)
C16	0.050 (2)	0.055 (2)	0.060 (2)	−0.0076 (18)	0.0073 (18)	−0.007 (2)
C15	0.052 (2)	0.059 (2)	0.046 (2)	−0.0061 (18)	−0.0012 (16)	−0.0006 (19)
C14	0.079 (3)	0.053 (2)	0.040 (2)	−0.011 (2)	0.0035 (19)	0.0030 (18)
O1	0.099 (2)	0.114 (3)	0.077 (2)	0.009 (2)	−0.0381 (18)	−0.0112 (18)
C13	0.054 (2)	0.067 (3)	0.057 (2)	−0.001 (2)	−0.0025 (18)	0.009 (2)
C12	0.062 (3)	0.050 (2)	0.077 (3)	−0.011 (2)	0.004 (2)	0.004 (2)
C11	0.054 (2)	0.081 (3)	0.051 (2)	−0.002 (2)	0.0008 (18)	0.006 (2)
C10	0.082 (3)	0.055 (2)	0.057 (2)	−0.001 (2)	0.004 (2)	−0.001 (2)
C9	0.050 (2)	0.059 (3)	0.076 (3)	−0.002 (2)	0.0022 (19)	0.011 (2)
C8	0.068 (3)	0.065 (3)	0.049 (2)	−0.011 (2)	−0.012 (2)	0.003 (2)
C7	0.058 (3)	0.098 (3)	0.062 (3)	0.007 (2)	0.002 (2)	0.020 (3)
C6	0.119 (4)	0.069 (3)	0.046 (2)	−0.012 (3)	−0.001 (2)	−0.004 (2)
C5	0.166 (6)	0.070 (3)	0.056 (3)	−0.010 (4)	0.017 (3)	−0.015 (2)
C4	0.072 (3)	0.076 (3)	0.072 (3)	0.018 (2)	0.008 (2)	0.020 (3)
C3	0.089 (3)	0.079 (3)	0.084 (3)	0.012 (3)	0.025 (3)	0.009 (3)
C2	0.143 (5)	0.075 (3)	0.079 (4)	0.014 (4)	0.046 (3)	−0.002 (3)
C1	0.091 (3)	0.072 (3)	0.102 (3)	0.000 (3)	−0.003 (3)	0.030 (3)

### Geometric parameters (Å, °)

**Table:**

N—C16	1.369 (4)	O1—C8	1.215 (4)
N—C24	1.379 (4)	C13—C9	1.363 (5)
N—C20	1.391 (4)	C13—H13	0.9300
C24—C21	1.380 (4)	C12—C9	1.401 (5)
C24—C17	1.430 (5)	C12—H12	0.9300
O3—C17	1.230 (4)	C11—C7	1.378 (5)
O2—C18	1.215 (4)	C11—H11	0.9300
C23—C19	1.382 (5)	C10—C3	1.388 (5)
C23—C22	1.400 (4)	C10—H10	0.9300
C23—C18	1.492 (5)	C9—C1	1.505 (5)
C22—C11	1.389 (5)	C7—C4	1.366 (5)
C22—C17	1.474 (5)	C7—H7	0.9300
C21—C15	1.399 (4)	C6—C5	1.372 (6)
C21—C18	1.462 (5)	C6—H6	0.9300
C20—C13	1.400 (4)	C5—C2	1.358 (6)
C20—C15	1.404 (5)	C5—H5	0.9300
C19—C4	1.377 (5)	C4—H4	0.9300
C19—H19	0.9300	C3—C2	1.376 (6)
C16—C12	1.338 (5)	C3—H3	0.9300
C16—H16	0.9300	C2—H2	0.9300
C15—C8	1.465 (5)	C1—H1A	0.9600
C14—C6	1.372 (5)	C1—H1B	0.9600
C14—C10	1.375 (5)	C1—H1C	0.9600
C14—C8	1.488 (5)		
			
C16—N—C24	130.0 (3)	C16—C12—C9	121.7 (4)
C16—N—C20	121.5 (3)	C16—C12—H12	119.2
C24—N—C20	108.5 (3)	C9—C12—H12	119.2
N—C24—C21	107.7 (3)	C7—C11—C22	120.1 (4)
N—C24—C17	126.5 (3)	C7—C11—H11	120.0
C21—C24—C17	125.7 (3)	C22—C11—H11	120.0
C19—C23—C22	119.3 (3)	C14—C10—C3	120.3 (4)
C19—C23—C18	119.2 (3)	C14—C10—H10	119.9
C22—C23—C18	121.4 (3)	C3—C10—H10	119.9
C11—C22—C23	119.2 (3)	C13—C9—C12	118.5 (4)
C11—C22—C17	119.4 (3)	C13—C9—C1	121.3 (4)
C23—C22—C17	121.4 (3)	C12—C9—C1	120.1 (4)
C24—C21—C15	109.4 (3)	O1—C8—C15	119.1 (4)
C24—C21—C18	119.8 (3)	O1—C8—C14	119.6 (3)
C15—C21—C18	130.5 (3)	C15—C8—C14	121.3 (3)
N—C20—C13	117.6 (3)	C4—C7—C11	120.9 (4)
N—C20—C15	108.3 (3)	C4—C7—H7	119.6
C13—C20—C15	134.2 (3)	C11—C7—H7	119.5
C23—C19—C4	120.9 (4)	C14—C6—C5	120.9 (5)
C23—C19—H19	119.5	C14—C6—H6	119.5
C4—C19—H19	119.5	C5—C6—H6	119.5
O2—C18—C21	123.4 (3)	C2—C5—C6	120.1 (5)
O2—C18—C23	120.6 (3)	C2—C5—H5	120.0
C21—C18—C23	116.0 (3)	C6—C5—H5	120.0
O3—C17—C24	123.1 (3)	C7—C4—C19	119.6 (4)
O3—C17—C22	121.8 (3)	C7—C4—H4	120.2
C24—C17—C22	115.1 (3)	C19—C4—H4	120.2
C12—C16—N	119.6 (3)	C2—C3—C10	119.5 (5)
C12—C16—H16	120.2	C2—C3—H3	120.3
N—C16—H16	120.2	C10—C3—H3	120.3
C21—C15—C20	106.1 (3)	C5—C2—C3	120.2 (5)
C21—C15—C8	131.6 (3)	C5—C2—H2	119.9
C20—C15—C8	122.3 (3)	C3—C2—H2	119.9
C6—C14—C10	119.0 (4)	C9—C1—H1A	109.5
C6—C14—C8	119.8 (4)	C9—C1—H1B	109.5
C10—C14—C8	121.2 (3)	H1A—C1—H1B	109.5
C9—C13—C20	121.1 (4)	C9—C1—H1C	109.5
C9—C13—H13	119.4	H1A—C1—H1C	109.5
C20—C13—H13	119.4	H1B—C1—H1C	109.5
			
C16—N—C24—C21	−178.7 (3)	C24—C21—C15—C20	−0.1 (4)
C20—N—C24—C21	−0.2 (3)	C18—C21—C15—C20	172.7 (3)
C16—N—C24—C17	0.7 (5)	C24—C21—C15—C8	179.2 (3)
C20—N—C24—C17	179.2 (3)	C18—C21—C15—C8	−8.0 (6)
C19—C23—C22—C11	0.7 (5)	N—C20—C15—C21	0.0 (4)
C18—C23—C22—C11	178.3 (3)	C13—C20—C15—C21	178.8 (4)
C19—C23—C22—C17	−177.9 (3)	N—C20—C15—C8	−179.4 (3)
C18—C23—C22—C17	−0.2 (5)	C13—C20—C15—C8	−0.6 (6)
N—C24—C21—C15	0.2 (4)	N—C20—C13—C9	0.4 (5)
C17—C24—C21—C15	−179.2 (3)	C15—C20—C13—C9	−178.3 (4)
N—C24—C21—C18	−173.5 (3)	N—C16—C12—C9	0.9 (5)
C17—C24—C21—C18	7.1 (5)	C23—C22—C11—C7	0.7 (5)
C16—N—C20—C13	−0.2 (4)	C17—C22—C11—C7	179.3 (3)
C24—N—C20—C13	−178.9 (3)	C6—C14—C10—C3	1.2 (5)
C16—N—C20—C15	178.8 (3)	C8—C14—C10—C3	−176.3 (3)
C24—N—C20—C15	0.1 (3)	C20—C13—C9—C12	0.0 (5)
C22—C23—C19—C4	−2.1 (5)	C20—C13—C9—C1	177.8 (3)
C18—C23—C19—C4	−179.7 (3)	C16—C12—C9—C13	−0.7 (5)
C24—C21—C18—O2	168.7 (3)	C16—C12—C9—C1	−178.5 (4)
C15—C21—C18—O2	−3.5 (6)	C21—C15—C8—O1	140.5 (4)
C24—C21—C18—C23	−9.5 (5)	C20—C15—C8—O1	−40.3 (5)
C15—C21—C18—C23	178.3 (3)	C21—C15—C8—C14	−43.1 (6)
C19—C23—C18—O2	5.7 (5)	C20—C15—C8—C14	136.1 (4)
C22—C23—C18—O2	−171.9 (3)	C6—C14—C8—O1	−27.8 (5)
C19—C23—C18—C21	−176.0 (3)	C10—C14—C8—O1	149.7 (4)
C22—C23—C18—C21	6.4 (4)	C6—C14—C8—C15	155.8 (3)
N—C24—C17—O3	−0.8 (5)	C10—C14—C8—C15	−26.7 (5)
C21—C24—C17—O3	178.5 (3)	C22—C11—C7—C4	−0.8 (6)
N—C24—C17—C22	−179.9 (3)	C10—C14—C6—C5	1.3 (5)
C21—C24—C17—C22	−0.7 (5)	C8—C14—C6—C5	178.8 (4)
C11—C22—C17—O3	−0.5 (5)	C14—C6—C5—C2	−2.9 (7)
C23—C22—C17—O3	178.1 (3)	C11—C7—C4—C19	−0.5 (6)
C11—C22—C17—C24	178.6 (3)	C23—C19—C4—C7	1.9 (6)
C23—C22—C17—C24	−2.8 (4)	C14—C10—C3—C2	−2.1 (6)
C24—N—C16—C12	177.9 (3)	C6—C5—C2—C3	2.0 (7)
C20—N—C16—C12	−0.5 (5)	C10—C3—C2—C5	0.5 (7)

### Hydrogen-bond geometry (Å, °)

**Table:**

*D*—H···*A*	*D*—H	H···*A*	*D*···*A*	*D*—H···*A*
C10—H10···O3^i^	0.93	2.45	3.305 (5)	152
C16—H16···O3	0.93	2.40	2.960 (5)	119

Symmetry codes: (i) −*x*+1, −*y*+1, −*z*+1.
